# The Impact of Hospital/Surgeon Volume on Acute Renal Failure and Mortality in Liver Transplantation: A Nationwide Cohort Study

**DOI:** 10.1371/journal.pone.0162992

**Published:** 2016-10-05

**Authors:** Chih-Wen Cheng, Fu-Chao Liu, Jr-Rung Lin, Yung-Fong Tsai, Hsiu-Pin Chen, Huang-Ping Yu

**Affiliations:** 1 Department of Anesthesiology, Chang Gung Memorial Hospital, Taoyuan, 333, Taiwan; 2 College of Medicine, Chang Gung University, Taoyuan, 333, Taiwan; 3 Clinical Informatics and Medical Statistics Research Center and Graduate Institute of Clinical Medicine, Chang Gung University, Taoyuan, 333, Taiwan; Imperial College London, UNITED KINGDOM

## Abstract

The aim of this study was to assess whether the case volume of surgeons and hospitals affects the rates of postoperative complications and survival after liver transplantation. This population-based retrospective cohort study included 2938 recipients of liver transplantation performed between 1998 and 2012, enrolled from the Taiwan National Health Insurance Research Database. They were divided into two groups, according to the cumulative case volume of their operating surgeons and the case volume of their hospitals. The duration of intensive care unit stay and post-transplantation hospitalization, postoperative complications, and mortality were analyzed. The results showed that, in the low and high case volume surgeons groups, respectively, acute renal failure occurred at the rate of 14.11% and 5.86% (p<0.0001), and the overall mortality rates were 19.61% and 12.44% (p<0.0001). In the low and high case volume hospital groups, respectively, acute renal failure occurred in 11% and 7.11% of the recipients (p = 0.0004), and the overall mortality was 18.44% and 12.86% (p<0.0001). These findings suggest that liver transplantation recipients operated on higher case volume surgeons or in higher case volume hospitals have a lower rate of acute renal failure and mortality.

## Introduction

Nowadays, liver transplantation is performed to treat for a variety of liver diseases such as acute hepatic failure, cholestatic disease, congenital biliary disease, cirrhosis, liver tumors, and metabolic diseases [1, 2]. The experience of the doctors or the care teams may be an important factor affecting the outcomes of complex surgical procedures. In colorectal cancer surgery, better outcomes were reported for patients treated in high -volume hospitals and by high -volume colorectal specialists [3, 4]. For pancreatic surgery, lower 1-year mortality was observed in higher volume hospitals [5, 6]. Perioperative complications were lower with high -volume surgeons and at high -volume centers after adult spinal deformity revision surgery [7].

However, the caseload of medical caregivers or facilities does not always affect outcomes. Hospital volume did not affect the mortality of preterm patent ductus arteriosus and biliary atresia [8, 9]. The survival rate after lung cancer surgery did not correlate with hospital case volume [10, 11]. In laparoscopic partial colectomy, low volume surgeons were observed to have similar outcomes compared to high volume surgeons [12]. It remains unknown whether hospital or surgeon case volume is related to the outcome of liver transplant patients. The goal of this nationwide cohort study was to assess whether case -volume of surgeons and hospitals had an impact on survival after liver transplantation. We therefore followed up the postoperative complication and mortality figures of patients receiving liver transplantation from 1998 to 2012 in Taiwan.

## Materials and Methods

### Study Population and Database

This was a retrospective cohort study and approved by the National Health Insurance Research Database (NHIRD) research committee (NHIRD-103-103) and the Institutional Review Board of the Chang Gung Medical Foundation.

The data was derived from the Taiwan NHIRD, which is an insurance claim database of Taiwan National Health Insurance launched in 1995 and, by 2014, covered 99.9% of the Taiwanese population. From 1998 onwards, the insurance registration files and original claim files by which patients, medical caregivers, and medical institutions could be identified have been encrypted and then sent to the National Health Research Institutes (NHRI) for setting up the database. The Bureau of National Health Insurance (BNHI) has collected claim data in a de-identified and computerized format and established the NHIRD. No consent was given since the data were analyzed anonymously. Before transferring data to scientists in Taiwan, NHRI encrypt the data again to protect the privacy of patients, medical caregivers, and medical institutions. Further, scientists intending to analyze data from NHRI have to sign a declaration of non-violation of privacy.

### Selection of Patients and Variables

**Fig 1:**
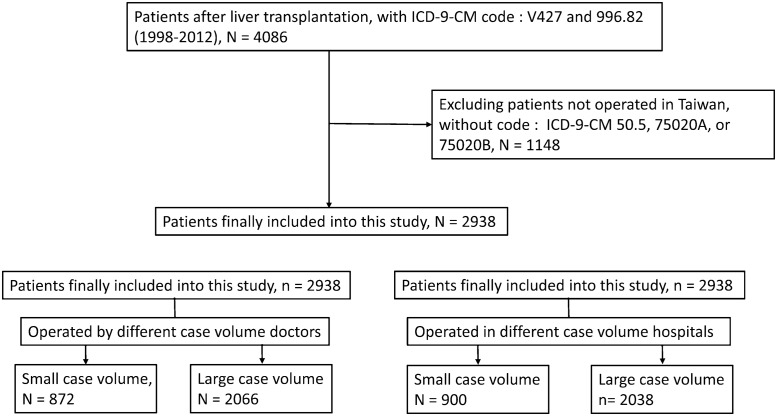
Flowchart of patient selection.

The flowchart of patient selection is shown in Fig 1.

Patients with the ICD-9-CM (International Classification of Diseases, Ninth Revision, Clinical Modification) codes V42.7 (liver replaced by transplant) and 996.82 (complications of transplanted liver) were identified from NHIRD data for the period between July 1998 and December 2012, and a total of 4086 patients were initially included in the study. Of these, 1148 patients were excluded because of not having undergone operation in Taiwan, or because they lacked ICD-9-CM 50.5, 75020A, or 75020B codes [13]. Finally, 2938 patients were included in this cohort study.

Preoperative comorbidity was identified from medical records of inpatient or outpatient departments using ICD-9-CM codes: hypertension (401–405), pulmonary disease (490–496), diabetes mellitus (250, A181), cerebrovascular disease (430–43, A291-A299), coronary heart disease (410–414, A279), liver cirrhosis (571.5, 571.2, 571.6), chronic kidney disease (585), hepatitis B (070.2, 070.3, V02.61, V02.69), and hepatitis C (070.41, 070.44, 070.51, 070.54, 070.7, V02.62).

### Defining Surgeon and Hospital Case Volumes

The methods for defining low and high case volume for surgeons and for hospitals are described as below. Surgeons were ranked according to their cumulative number of liver transplantations performed, and were divided into three groups. The group with the most cases (one-third of all surgeons) was defined as the high case volume surgeons group, and the others (two-thirds) were defined as the low case volume surgeons group [14]. The cutoff number was 100, which meant that surgeons who had performed more than 100 liver transplantation procedures were considered high volume surgeons. Hospitals were ranked according to the cumulative number of liver transplantation cases, and were divided into three groups. The group with the most cases (one-third of all hospitals) was defined as the high case volume hospitals group, and the others (two-thirds) were defined as the low case volume hospitals group [14]. The cutoff number of cumulative liver transplantation procedures for defining case volume in the hospital was 300.

### Outcomes

The primary outcome was mortality rates after liver transplantation. Others were the length of intensive care unit (ICU) stay and hospitalization, bacteremia, pneumonia, bleeding, and acute renal failure after liver transplantation. Patients were identified by the postoperative occurrence of the ICD-9-CM code of bacteremia, pneumonia, and bleeding. Acute renal failure after liver transplantation was defined as the patient receiving renal replacement therapy from the end of liver transplantation surgery to discharge from the hospital. Patients were excluded if on renal replacement therapy prior to liver transplantation with ICD-9-CM codes 38.95, 39.27, 39.42, 39.95, 39.43 or 54.98. Recipients with the postoperative ICD-9-CM codes 584, 585, V451 or V56 were regarded as having acute renal failure. Death was defined as the ending of national health insurance or with a death code in the records [13]. The total numbers of renal replacement therapies were calculated.

### Statistical Analysis

The SAS statistical software, version 9.3, (SAS institute Inc, Cary, NC) was used to evaluate our data. A p-value less than 0.05 was considered statistically significant. The T test, chi-squared test, or Fisher’s exact test was used to analyze demographic data, coexisting underlying disease, length of medical inpatient service (such as length of ICU stay and hospitalization), and postoperative complications.

During the post-transplantation period, Kaplan-Meier estimates with the log-rank test were used for comparing survival rates between groups. When analyzing mortality, patients were followed up after liver transplantation until either death or censoring. Mortality risks were evaluated by multivariate logistic regression analysis.

## Results

### Mortality

This cohort study analyzed 2938 patients. The cumulative mortality rates for all the liver transplantation recipients was 1.1% at 30 days, 2.8% at 3 -months, 7.7% at 1 -year, 14.6% overall.

### Volume Effect of Surgeons

Low case volume surgeons were found to have operated on 872 patients, while the other 2066 patients had been operated on by high case volume surgeons. The demographic analysis is shown in Table 1.

**Table 1:** Demography of liver transplantation recipients operated on by low and high case volume surgeons.

	Low case volume surgeons (n = 872)	High case volume surgeons (n = 2066)	P Value
	Mean(SD) / n (%)	Mean(SD) / n (%)	
Age^☨^	46.10 (17.23)	46.56 (17.87)	0.5171
Gender			0.5824
Female	252 (28.90)	618 (29.91)	
Male	620 (71.10)	1448 (70.09)	
Pre-operative clinical parameters			
Hypertension	177 (20.30)	423 (20.47)	0.9138
Pulmonary diseases	136 (15.60)	284 (13.75)	0.1906
Diabetes mellitus	169 (19.38)	445 (21.54)	0.1886
Cerebrovascular disease	33 (3.78)	58 (2.81)	0.1626
Coronary heart disease	58 (6.65)	146 (7.07)	0.6857
Liver cirrhosis	739 (84.75)	1745 (84.46)	0.8452
Hepatitis B	434 (49.77)	982 (47.53)	0.2671
Hepatitis C	193 (22.13)	469 (22.70)	0.7364

^☨^ Values are mean and standard deviation.

SD, standard deviation.

T-test or Chi-square test were used to examine the differences in the demographic characteristics of liver transplant patients between the small and large case volume doctors.

Recipients operated on by low case volume surgeons stayed in the ICU for a longer period than those operated on by high case volume surgeons (*p*<0.05). However, the length of hospital stay after liver transplantation was similar for both groups (Table 2). Postoperatively, the incidence of bacteremia, pneumonia, and bleeding were similar between low and high case volume surgeon groups (*p*>0.05). More acute renal failure was observed in the low case volume surgeon groups (*p* = 0.0004).

**Table 2:** Outcomes of liver transplantation recipients operated on by low and high case volume surgeons.

	Low case volume surgeons (n = 872)	High case volume surgeons(n = 2066)	P Value
	Mean(SD) / n (%)	Mean(SD) / n (%)	
ICU stay, (days) ^☨^	17.36 (22.13)	15.45 (14.60)	0.0199*
Hospital stay, (days) ^☨^	50.17 (39.53)	47.82 (33.09)	0.1226
Bacteremia	59 (6.77)	106 (5.13)	0.0786
Pneumonia	32 (3.67)	54 (2.61)	0.1208
Postoperative bleeding	44 (5.05)	107 (5.18)	0.8812
Acute Renal Failure	123 (14.11)	121 (5.86)	<0.0001*

^☨^ Values are mean and standard deviation.

*P value < 0.05.

ICU stay, intensive care unit stay. SD, standard deviation.

T-test or Chi-square test were used to examine the differences in the outcomes of liver transplant patients between the small and large case volume doctors.

As shown in Table 3, mortality rates were noted at 30 -days, at 3 -months, at 1 -year and overall; recipients in the low case -volume surgeon group had a higher mortality rate than those in the high case -volume surgeon group (*p*< 0.0001).

**Table 3:** Mortality rate of liver transplantation recipients operated on by low and high case volume surgeons.

	Low case volume surgeons (n = 872)		High case volume surgeons (n = 2066)		P Value
	n (%)	Median	n (%)	Median	
Mortality (30-days)	25 (2.87)	17	9 (0.45)	18	<0.0001*
Mortality (3-months)	57 (6.54)	35	25 (1.21)	43	<0.0001*
Mortality (1-year)	106 (12.16)	76.5	120 (5.81)	190.5	<0.0001*
Mortality (Overall)	171 (19.61)	398.5	257 (12.44)	74	<0.0001*

*P value < 0.05.

The Log–rank test were used to examine the differences in the mortality rate of liver transplantation recipients operated by small and large case volume doctors.

**Fig 2:**
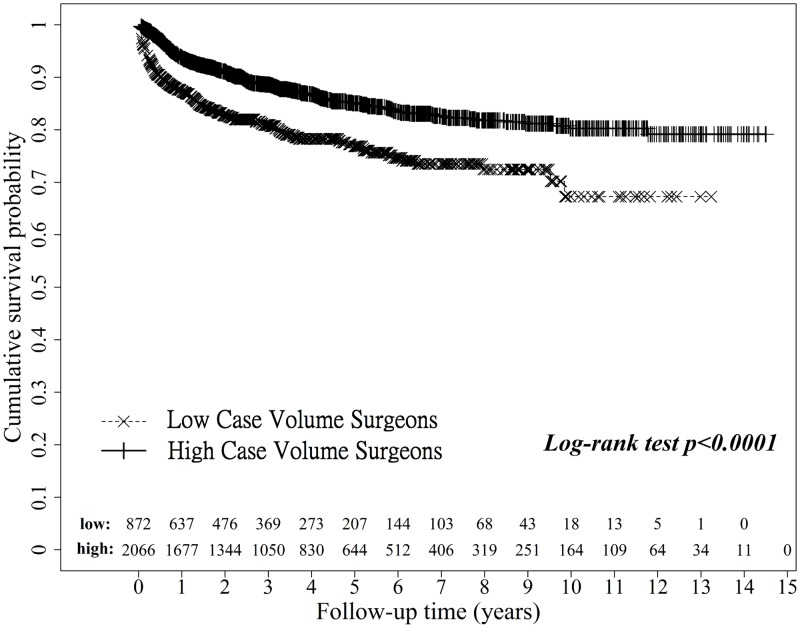
Overall mortality when operated on by high and low case volume surgeons.

The unadjusted Kaplan-Meier survival analysis, shown in Fig 2, revealed a one-year survival rate of 87.84% in the low case volume surgeon group and of 94.19% in the high case volume surgeon group. The overall survival was 80.39% in the low case volume surgeon group as compared to 87.56% in the high case volume surgeon group. There is significant association between chronic renal failure before liver transplant and acute renal failure after liver transplant surgery by surgeon groups respectively. The percent of patient with chronic renal failure before surgery and acute renal failure after surgery are 42.9% in high case volume surgeon group and 37.5% in low case volume surgeon group, and without chronic renal failure before surgery and with acute renal failure after surgery are 11.6% in high case volume surgeon group and 20.4% in low case volume surgeon group, high case volume surgeon (Chi-square test p-value < .0001) and low case volume surgeon (Chi-square test p-value = 0.0421) respectively. The average numbers of renal replacement therapies are no different between surgeon groups (p-value = 0.6639).

### Volume Effect of Hospitals

The low and high case volume hospital groups comprised 900 and 2038 operated patients, respectively. The demographic information for these two groups is shown in Table 4.

**Table 4:** Demography of liver transplantation recipients operated on in low and high case volume hospitals.

	Low case volume hospitals (n = 900)	High case volume hospitals (n = 2038)	P Value
	Mean(SD) / n (%)	Mean(SD) / n (%)	
Age^☨^	48.86 (14.69)	45.34 (18.75)	<0.0001*
Gender			
Female	224 (24.89)	646 (31.70)	0.0002*
Male	676 (75.11)	1392 (68.30)	
Pre-operative clinical parameters			
Hypertension	222 (24.67)	378 (18.55)	0.0001*
Pulmonary diseases	153 (17.00)	267 (13.10)	0.0054*
Diabetes mellitus	192 (21.33)	422 (20.71)	0.7001
Cerebrovascular disease	35 (3.89)	56 (2.75)	0.0998
Coronary heart disease	72 (8.00)	132 (6.48)	0.1344
Liver cirrhosis	770 (85.56)	1714 (84.10)	0.3150
Hepatitis B	485 (53.89)	931 (45.68)	<0.0001*
Hepatitis C	205 (22.78)	457 (22.42)	0.8324

^☨^ Values are mean and standard deviation.

*P value < 0.05.

SD, standard deviation.

T-test or Chi-square test were used to examine the differences in the demographic characteristics of liver transplant patients between the small and large case volume hospitals.

Patients who underwent liver transplantation in low case volume hospitals stayed in the ICU and in hospital for a shorter duration than those in high case volume hospitals (Table 5). The incidence of bacteremia, pneumonia, and bleeding after liver transplantation was similar between the low and high volume hospital groups. Acute renal failure during the postoperative period occurred more frequently in the low volume hospital group.

**Table 5:** Outcomes of liver transplantation recipients operated on in low and high case volume hospitals.

	Low case volume hospitals(n = 900)	High case volume hospitals (n = 2038)	P Value
	Mean(SD) / n (%)	Mean(SD) / n (%)	
ICU stay, (days) ^☨^	11.69(13.15)	17.94(18.46)	<0.0001*
Hospital stay, (days) ^☨^	38.63 (29.81)	52.89(36.40)	<0.0001*
Bacteremia	49 (5.44)	116 (3.95)	0.7883
Pneumonia	24 (2.67)	62 (3.04)	0.5778
Postoperative bleeding	36 (4.00)	115 (5.64)	0.0630
Acute Renal Failure	99 (11.00)	145 (7.11)	0.0004*

^☨^ Values are mean and standard deviation.

*P value < 0.05.

ICU stay, intensive care unit stay. SD, standard deviation.

T-test or Chi-square test were used to examine the differences in the outcomes of liver transplant patients between the small and large case volume hospitals.

**Fig 3:**
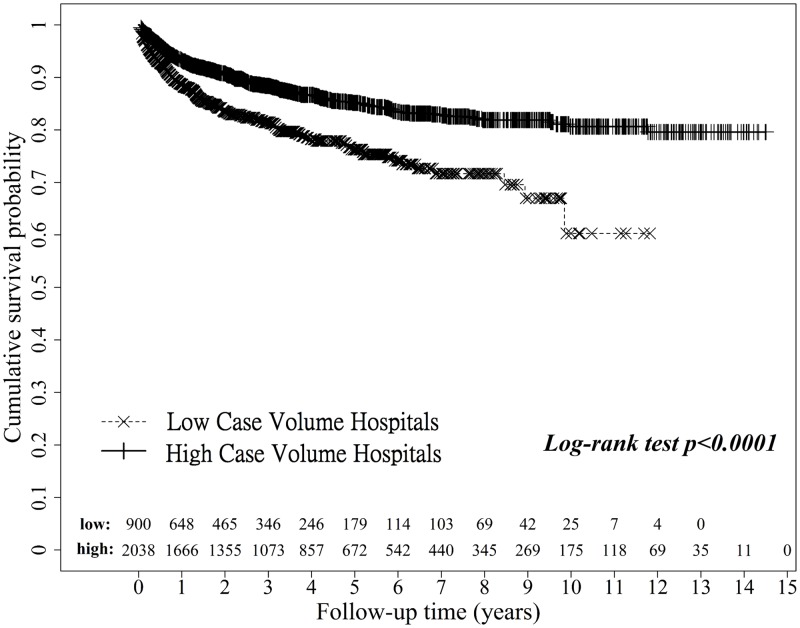
Overall mortality when operated on in high and low case volume hospitals.

As shown in Table 6, the mortality in the low and high volume hospital groups followed up at different times was 2.00% vs. 0.79% at 30 days (p = 0.0045), 4.56% vs. 2.01% at 3 months (p = 0.0001), 10.56% vs. 6.43% at 1 year (p<0.0001), and 18.44% vs. 12.86% overall (p< 0.0001). Thus, higher mortality was observed in low volume hospitals. The unadjusted Kaplan-Meier survival analysis is shown in Fig 3. There is significant association between chronic renal failure before liver transplant and acute renal failure after liver transplant surgery by hospital groups respectively. The percent of patient with chronic renal failure before surgery and acute renal failure after surgery are 36.4% in high case volume hospital and 46.9% in low case volume hospital, and without chronic renal failure before surgery and with acute renal failure after surgery are 12.7% in high case volume hospital and 17.5% in low case volume hospital, high case volume hospital (Fisher exact test p-value = 0.0006) and low case volume hospital (Chi-square test p-value < .0001) respectively. The average numbers of renal replacement therapies are no different between hospital groups (p-value = 0.8912).

**Table 6:** Mortality rate of liver transplantation recipients operated on in low and high case volume hospitals.

	Low case volume hospitals(n = 900)		High case volume hospitals (n = 2038)		P Value
	n (%)	Median	n (%)	Median	
Mortality (30-days)	18 (2.00)	19.5	16 (0.79)	15	0.0045*
Mortality (3-months)	41 (4.56)	35	41 (2.01)	36	0.0001*
Mortality (1-year)	95 (10.56)	108	131 (6.43)	148	<0.0001*
Mortality (Overall)	166 (18.44)	273	262 (12.86)	365.5	<0.0001*

*P value < 0.05.

The Log–rank test were used to examine the differences in the mortality rate of liver transplantation recipients operated by small and large case volume hospitals.

## Discussion

It was observed that outcomes after transplantation surgery correlated with case volume of the surgeon and the hospital [15, 16]. The mortality at 30 days, at 3 months, at 1 year, and overall, was significantly lower when recipients were operated on by surgeons, or in hospitals, with high case volumes. In addition, the incidence of postoperative acute renal failure was lower if surgery was performed by surgeons, or in hospitals, with high case volumes.

High surgeon case volume has been reported to be a good survival factor in many surgical procedures, such as hepatectomy, colorectal cancer surgery [3, 17, 18]. Liver transplantation is a complex surgical procedure. Previous studies have shown that the steep learning curve for liver transplantation may affect the recipients’ outcome [19, 20] and less experienced surgeons had higher short-term postoperative complication rates and lower 1-year survival rates. The 1-year survival rates improved as the surgeons’ experience increased, but reached a plateau despite further cumulated experience. Therefore, at this stage, the mortality rate would be related to the surgeons’ experience.

High surgical volume of hospitals was also an indicator for a lower complication rate and a factor for better survival in many surgical procedures [5, 15, 21, 22]. High volume hospitals had more experienced healthcare staff and specialized units providing better care. More skilled specialists for perioperative care, as well as more experienced nurses and ICU staff might play an important role [17]. We also found that high case volume hospitals had lower rates of acute renal failure and mortality.

The incidence of postoperative acute renal failure was higher in patients who underwent liver transplantation by low volume surgeons and in low volume hospitals. Patients who encountered acute renal failure after liver transplantation also had increased mortality rates [13, 23]. Consistent with these findings, our study also found that liver transplantation recipients operated on by high case volume surgeons or operated in high case volume hospitals have a lower rate of acute renal failure and mortality. Renal replacement therapy is usually initiated in the setting of medically refractory hyperkalemia, acidosis, or volume overload or if uremic symptoms developed [24].

This retrospective cohort study has several limitations. First, the NHIRD is an insurance claim database, so clinical data is not collected. The preoperative model for end-stage liver disease (MELD) score, Child-Pugh score, amount of intraoperative blood loss, blood pressure, type and amount of transfusion, urine output, cold and warm ischemia time of the graft, and amount of postoperative blood loss could not be traced and analyzed. Second, the data was from the ICD-9-CM code. Taiwan National Insurance Bureau verifies the code periodically, but coding faults may occur due to human error. Third, our cohort covered a time -span of 14.5 years and, during this period, medical advances could have affected the mortality rates [13]. Finally, this study only reveals the relationship between case volume and mortality after liver transplantation; however, the definite reasons for mortality remain unclear.

## Conclusion

Our study suggests that surgeons’ and hospitals’ case volume are related to patients’ mortality after liver transplantation. In this cohort study, patients who underwent liver transplantation by high case volume surgeons or in high case volume hospitals had a better survival rate. Centralization of liver transplantation surgery to high case volume surgeons and hospitals may decrease mortality.

## Supporting Information

S1 File Numbers of liver transplant patients sorted by surgeons. (XLSX)

S2 File Numbers of liver transplant patients sorted by hospitals. (XLSX)

## References

[1] Adam R, Karam V, Delvart V, O'Grady J, Mirza D, Klempnauer J, et al Evolution of indications and results of liver transplantation in Europe. A report from the European Liver Transplant Registry (ELTR). J Hepatol. 2012;57:675–688. 10.1016/j.jhep.2012.04.015 22609307

[2] Fox AN, Brown RS Jr. Is the patient a candidate for liver transplantation? Clin Liver Dis. 2012;16:435–448. 10.1016/j.cld.2012.03.014 22541708

[3] Archampong D, Borowski D, Wille-Jorgensen P, Iversen LH. Workload and surgeon's specialty for outcome after colorectal cancer surgery. Cochrane Database Syst Rev. 2012;3:CD005391 10.1002/14651858.CD005391.pub3 22419309 PMC12076000

[4] Billingsley KG, Morris AM, Dominitz JA, Matthews B, Dobie S, Barlow W, et al Surgeon and hospital characteristics as predictors of major adverse outcomes following colon cancer surgery: understanding the volume-outcome relationship. Arch Surg. 2007;142:23–31; discussion 2. 10.1001/archsurg.142.1.23 17224497

[5] Alsfasser G, Leicht H, Gunster C, Rau BM, Schillinger G, Klar E. Volume-outcome relationship in pancreatic surgery. Br J Surg. 2016;103:136–143. 10.1002/bjs.9958 26505976

[6] Hata T, Motoi F, Ishida M, Naitoh T, Katayose Y, Egawa S, et al Effect of hospital volume on surgical outcomes after pancreaticoduodenectomy: A systematic review and meta-analysis. Ann Surg. 2016;263:664–672. 10.1097/SLA.0000000000001437 26636243

[7] Paul JC, Lonner BS, Goz V, Weinreb J, Karia R, Toombs CS, et al Complication rates are reduced for revision adult spine deformity surgery among high-volume hospitals and surgeons. Spine J. 2015;15:1963–1972. 10.1016/j.spinee.2015.04.028 25937293

[8] Schreiber RA, Barker CC, Roberts EA, Martin SR, Canadian Pediatric Hepatology Research G. Biliary atresia in Canada: the effect of centre caseload experience on outcome. J Pediatr Gastroenterol Nutr. 2010;51:61–65. 10.1097/MPG.0b013e3181d67e5e 20543720

[9] Michihata N, Matsui H, Fushimi K, Yasunaga H. Association between hospital volume and mortality of preterm patent ductus arteriosus. Pediatr Int. 2016 10.1111/ped.13008 27062220

[10] Sioris T, Sihvo E, Sankila R, Salo J. Effect of surgical volume and hospital type on outcome in non-small cell lung cancer surgery: a Finnish population-based study. Lung Cancer. 2008;59:119–125. 10.1016/j.lungcan.2007.07.020 17825951

[11] Osada H, Yamakoshi E. Hospital volume and surgical outcomes of lung cancer in Japan. Gen Thorac Cardiovasc Surg. 2007;55:360–365. 10.1007/s11748-007-0114-x 17937049

[12] Donkervoort SC, Dijksman LM, Versluis PG, Clous EA, Vahl AC. Surgeon's volume is not associated with complication outcome after laparoscopic cholecystectomy. Dig Dis Sci. 2014;59:39–45. 10.1007/s10620-013-2885-5 24081642

[13] Chen HP, Tsai YF, Lin JR, Liu FC, Yu HP. Incidence and outcomes of acute renal failure following liver transplantation: a population-based cohort study. Medicine (Baltimore). 2015;94:e2320 10.1097/MD.0000000000002320 26717368 PMC5291609

[14] Nathan H, Cameron JL, Choti MA, Schulick RD, Pawlik TM. The volume-outcomes effect in hepato-pancreato-biliary surgery: hospital versus surgeon contributions and specificity of the relationship. J Am Coll Surg. 2009;208:528–538. 10.1016/j.jamcollsurg.2009.01.007 19476786

[15] Weng SF, Chu CC, Chien CC, Wang JJ, Chen YC, Chiou SJ. Renal transplantation: relationship between hospital/surgeon volume and postoperative severe sepsis/graft-failure. a nationwide population-based study. Int J Med Sci. 2014;11:918–924. 10.7150/ijms.8850 25013372 PMC4081314

[16] Hayanga JA, Lira A, Vlahu T, D'Cunha J, Hayanga HK, Girgis R, et al Procedural volume and survival after lung transplantation in the United States: the need to look beyond volume in the establishment of quality metrics. Am J Surg. 2016;211:671–676. 10.1016/j.amjsurg.2015.12.010 26830718

[17] Chang CM, Yin WY, Wei CK, Lee CH, Lee CC. The combined effects of hospital and surgeon volume on short-term survival after hepatic resection in a population-based study. PLoS One. 2014;9:e86444 10.1371/journal.pone.0086444 24466102 PMC3899267

[18] Renzulli P, Lowy A, Maibach R, Egeli RA, Metzger U, Laffer UT. The influence of the surgeon's and the hospital's caseload on survival and local recurrence after colorectal cancer surgery. Surgery. 2006;139:296–304. 10.1016/j.surg.2005.08.023 16546492

[19] Li C, Mi K, Wen T, Yan L, Li B, Yang J, et al A learning curve for living donor liver transplantation. Dig Liver Dis. 2012;44:597–602. 10.1016/j.dld.2012.01.016 22387283

[20] Marin-Gomez LM, Tinoco-Gonzalez J, Alamo-Martinez JM, Suarez-Artacho G, Bernal-Bellido C, Serrano-Diaz-Canedo J, et al Impact of the learning curve on the outcome of domino liver transplantation. Transplant Proc. 2014;46:3092–3094. 10.1016/j.transproceed.2014.09.176 25420831

[21] Albornoz CR, Cordeiro PG, Hishon L, Mehrara BJ, Pusic AL, McCarthy CM, et al A nationwide analysis of the relationship between hospital volume and outcome for autologous breast reconstruction. Plast Reconstr Surg. 2013;132:192e–200e. 10.1097/PRS.0b013e31829586c1 23897346

[22] Edwards EB, Roberts JP, McBride MA, Schulak JA, Hunsicker LG. The effect of the volume of procedures at transplantation centers on mortality after liver transplantation. N Engl J Med. 1999;341:2049–2053. 10.1056/NEJM199912303412703 10615076

[23] Yalavarthy R, Edelstein CL, Teitelbaum I. Acute renal failure and chronic kidney disease following liver transplantation. Hemodial Int. 2007;11 Suppl 3:S7–S12. 10.1111/j.1542-4758.2007.00223.x 17897111

[24] Wald R, Adhikari NK, Smith OM, Weir MA, Pope K, Cohen A, et al Comparison of standard and accelerated initiation of renal replacement therapy in acute kidney injury. Kidney Int. 2015;88:897–904. 10.1038/ki.2015.184 26154928

